# Selective CO_2_ hydrogenation to formic acid on Cu_55_ and Cu_13_@Ni_42_ nanoclusters: a DFT and artificial bee colony optimization study

**DOI:** 10.1039/d5na01067e

**Published:** 2026-03-12

**Authors:** Norah O. Alotaibi, Shatha M. Alruwaythi, Heider A. Abdulhussein

**Affiliations:** a Chemistry Department, Faculty of Science, King Abdulaziz University 21589 Jeddah Saudi Arabia; b Department of Chemistry, Faculty of Science, University of Kufa Najaf Iraq haydera.abdulhussein@uokufa.edu.iq; c College of Engineering, University of Warith Al-Anbiyaa Kerbala Iraq

## Abstract

The selective hydrogenation of CO_2_ to formic acid is a promising route for carbon utilization and green hydrogen storage, yet the lack of highly active and selective nanocatalysts limits its practical deployment. In this work, we combine global structural optimization using the artificial bee colony (ABC) algorithm with density functional theory (DFT) simulations to systematically investigate the catalytic behavior of monometallic Cu_55_ and bimetallic core–shell Cu_13_@Ni_42_ nanoparticles. Global minima identified *via* the ABC-DFT framework reveal that both clusters adopt icosahedral-derived geometries but exhibit markedly different electronic structures and binding characteristics. Comprehensive adsorption analyses of key intermediates (
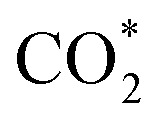
, H*, HCOO*, COOH*, and HCOOH*) show that Ni incorporation substantially strengthens CO_2_ activation, H_2_ dissociation, and intermediate stabilization. The Cu_13_@Ni_42_ cluster exhibits significantly more exothermic adsorption energies—most notably for CO_2_ (−0.924 eV) and CO (−3.745 eV), along with a thermodynamically more favorable formate pathway compared to Cu_55_. Thermochemical profiling confirms that the rate-determining hydrogenation step (HCOO* → HCOOH*) is energetically more accessible on Cu_13_@Ni_42_ (−0.709 eV) than on Cu_55_ (−0.470 eV), indicating higher catalytic efficiency. Density of states (DOS) analysis reveals strong 3d–3d orbital hybridization between Cu and Ni, which shifts the d-band center and enhances reactivity. Overall, the results establish the Cu_13_@Ni_42_ core–shell nanocluster as a superior candidate for selective CO_2_-to-formic acid conversion, offering improved thermodynamics, stronger CO_2_ activation, and more favorable electronic properties compared with monometallic Cu_55_.

## Abstract 2

DFT-guided design shows a bimetallic core–shell nanocatalyst outperforms a monometallic one for CO_2_ hydrogenation to formic acid, enabling stronger adsorption, better activation, and improved catalytic efficiency.

## Graphical abstract

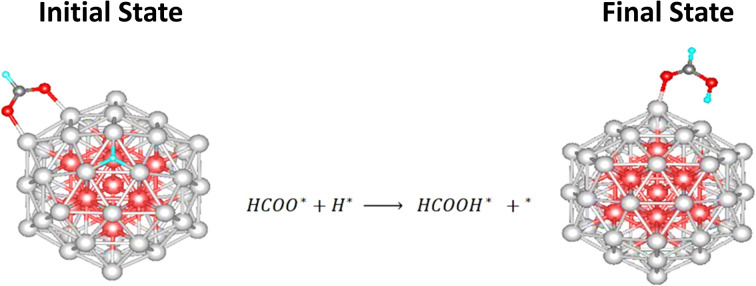

## 1. Introduction

The increasing global reliance on fossil fuels for energy generation is driving significant environmental challenges, highlighting the need for sustainable alternatives such as hydrogen, the most abundant element in the universe, that has gained growing interest due to its potential to reduce ecological impact.^1,2^ From a chemistry perspective, CO_2_ can be converted into value-added hydrocarbons and fuels (including HCOOH, CH_4_, CH_3_OH, among others) *via* carbon dioxide electrochemical reduction reaction (CO_2_ER).^3–5^ Although CO_2_ is an abundant and low-cost carbon source, its high thermodynamic stability necessitates the use of highly electrochemically conductive materials to facilitate its activation and conversion.^6^

The electrochemical conversion of CO_2_ to formic acid (HCOOH) has been well documented experimentally in the literature.^7,8^ A thorough understanding of the microscopic mechanisms governing CO_2_ hydrogenation is essential to establish direct hydrogenation as a cost-effective, environmentally sustainable, and safe method for formic acid production.^9,10^ Recent studies have revealed unique electronic and catalytic properties in size-selected subnanometer transition metal clusters.^11–14^ Catalysts incorporating small nanoclusters have demonstrated versatility across a broad spectrum of catalytic reactions.^15^ With the ability to control the size of nanocatalysts, both the physical and chemical properties could be tuned, providing better reaction efficiency.^16^

The design and development of electrocatalysts with low overpotentials and high selectivity for CO_2_ electrochemical reduction (CO_2_ER) have been extensively investigated through both experimental and theoretical studies.^14,17–20^ Nanostructured materials, characterized by their high surface area, offer a greater number of active sites compared to their bulk counterparts, thereby enhancing catalytic performance.^21^ Consequently, the engineering of nanostructured catalysts is widely regarded as an effective strategy for improving electrocatalytic activity.^22^ Among various metals, copper (Cu) has garnered significant attention due to its abundance and its capability to produce a range of products including CO, formic acid (HCOOH), and other hydrocarbons *via* CO_2_ER.^23,24^ However, the practical application of monometallic Cu catalysts is limited by their relatively high overpotentials and their pronounced activity toward the competing hydrogen evolution reaction (HER).^25^ This has led to growing interest in bimetallic nanocatalysts, which often exhibit enhanced catalytic properties compared to their monometallic counterparts.^26–29^ In particular, Cu-based bimetallic nanoalloys have been widely studied for their structural and electronic characteristics.^30–34^ Theoretical investigations have shown that copper–nickel (Cu–Ni) clusters possess promising adsorption capabilities for CO_2_.^35^ Specifically, studies on 55-atom clusters both Ni-doped and core–shell configurations have provided valuable insights into the interaction between CO_2_ molecules and Cu–Ni cluster surfaces.^36^

Bimetallic Cu–Ni nanoparticles have emerged as highly active and versatile catalysts, playing a central role in a range of industrially relevant reactions such as methane decomposition,^37^ ethanol steam reforming,^38^ and water gas shift reactions.^39^ Furthermore, CuNi catalysts can produce formic acid efficiently from the hydrogenation of CO_2_ to formic acid, an emerging hydrogen carrier medium.^40^ From a synthetic chemistry standpoint, bimetallic Cu–Ni core–shell alloys are typically prepared *via* the co-reduction of Cu and Ni precursors through heterogeneous nucleation. This process exploits the difference in standard reduction potentials between Cu^2+^ (0.337 V) and Ni^2+^ (0.257 V), whereby Cu is reduced preferentially to form the core, followed by Ni deposition to form the outer shell.^41^ Among these nanostructures, the Cu_13_@Ni_42_ core–shell cluster represents a particularly promising candidate, not only due to its synthetic feasibility but also its catalytic potential. Theoretical studies have shown that this cluster exhibits a strongly exothermic excess energy (*E*_exc_), along with robust CO_2_ adsorption capabilities, enabling both activation and subsequent transformation of CO_2_*via* dissociation and hydrogenation pathways.^42^

To the best of our knowledge, global optimization studies of Cu_55_ and Cu–Ni nanoclusters in the context of CO_2_ hydrogenation to formic acid have not yet been reported. In this study, we perform an in-depth theoretical analysis of the structural and thermodynamic characteristics of monometallic Cu_55_ and bimetallic core–shell Cu_13_@Ni_42_ clusters, aiming to computationally identify the most effective catalyst for selective CO_2_-to-formic acid conversion. This investigation addresses a significant gap in the literature and emphasizes the promising role of these nanoclusters as selective and efficient catalysts for sustainable chemical processes.

Although CO_2_ adsorption and activation on Cu–Ni clusters have been previously investigated, most notably in ref. 42, the scope of the present work is fundamentally different. Ref. 42 primarily focused on identifying stable Cu–Ni cluster motifs and establishing structure–property relationships governing CO_2_ adsorption and activation. In contrast, the present study extends beyond adsorption thermodynamics to explicitly address reaction-level catalysis, including the adsorption of key reaction intermediates, construction of complete hydrogenation pathways, and identification of kinetically and thermodynamically relevant steps toward formic acid formation. By mapping full reaction networks rather than isolated adsorption events, this work demonstrates that clusters exhibiting similar CO_2_ activation behavior may display markedly different reaction energetics and pathway selectivity. These results reveal that CO_2_ activation alone is not a sufficient descriptor for catalytic performance and underscore the importance of reaction intermediates and local atomic environments in determining catalytic activity and selectivity on Cu–Ni clusters.

## 2. Computational details

### 2.1. Model

**Fig. 1:**
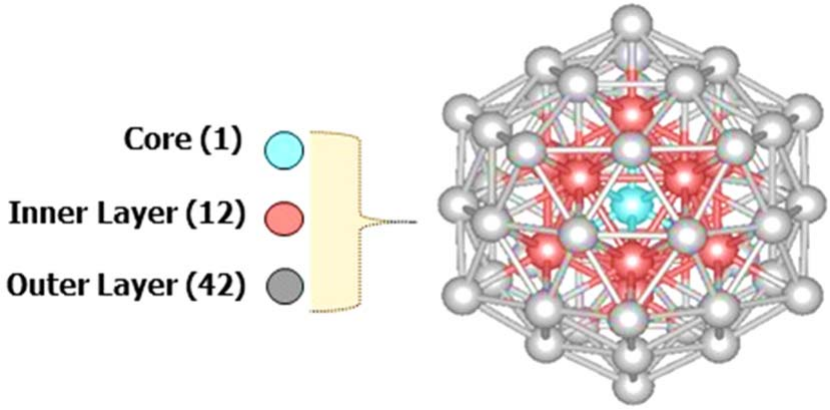
DFT-optimized geometry of the 55-atom icosahedron structure.

The 55-atom icosahedral cluster is a well-studied metallic cluster that has drawn considerable interest.^43–46^ Its stability is attributed to the formation of a closed-shell Mackay icosahedron consisting of 55 atoms with *I*_h_ symmetry.^47^ As shown in Fig. 1, this structure has a central atom surrounded by two shells: the first shell contains 12 atoms (inside the cluster), while the second shell contains the remaining 42 atoms (the outer layer of the cluster).

### 2.2. Cluster exploration using the DFT-ABC method

A key aspect of cluster research is efficiently locating the global minimum (GM), as experiments on clusters are typically performed at low temperatures where the GM configuration predominates. However, conducting a thorough GM search is notoriously difficult. For a cluster with N structural units, the potential energy surface (PES) has 3N degrees of freedom, and the number of local minima (LMs) increases exponentially with N.^48–56^ This difficulty arises from the highly complex and uneven distribution of the vast number of LMs. The PES is locally rugged, making it nearly impossible to explore thoroughly using conventional computer simulations for large clusters.^49,57^

In 2005, computer scientist Karaboga introduced the artificial bee colony (ABC) algorithm, a nature-inspired optimization technique based on swarm intelligence.^58^ The algorithm mimics the foraging behavior of honey bee colonies, where individual bees are assigned specific roles in searching for the most nutrient-rich nectar sources. The ABC computational model involves three types of bees: employed bees, onlooker bees, and scout bees. Each bee evaluates the quality of nectar (analogous to a solution) as it is discovered and can share this information with others. Communication methods like the waggle dance are essential for feedback and decision-making within the bee colony. Employed bees utilize information obtained from other bees to search for new nectar sources. Onlooker bees, guided by the knowledge shared by employed bees, concentrate their efforts around areas with high-quality nectar. In contrast, scout bees venture into unexplored regions, discarding low-quality sources based on input from both employed and onlooker bees. This iterative process of exploration enables the ABC algorithm to effectively address complex optimization challenges, such as sampling the potential energy surface (PES).

To perform a comprehensive search for the global minimum (GM) structures of clusters, we employed the ABC method as implemented in the ABCluster program^59,60^ utilizing an interface to the Vienna *Ab initio* Simulation Package (VASP)^61–64^ to simulate Cu_55_ and core–shell Cu_43_Ni_12_ nanoclusters. This approach effectively generates GM geometries using the nature-inspired artificial bee colony (ABC) algorithm. The search requires several key parameters: a large population of trial solutions (in our case, 500), a maximum of 1000 generations (resulting in over 50 000 possible atomic configurations), a scout limit (set to 5), the size of the nanoparticles (N), the estimated maximum coordinate value (*L*). All remaining parameters were maintained at their default settings. Additional details are provided in the referenced literature.^60^

DFT-based atomistic simulations have emerged as a widely used approach for elucidating the structural and electronic behavior of crystalline materials, spanning systems from discrete molecules to extended solids.^65–70^ Gamma-point, spin-polarized, periodic DFT calculations are carried out using the revised Perdew–Burke–Ernzerhof (rev-PBE) exchange–correlation functional, as implemented in the Vienna *Ab initio* Simulation Package (VASP).^63,64,71,72^ Core electrons were treated using the projector augmented-wave (PAW) pseudopotentials.^73,74^ Plane-wave basis sets (with a kinetic energy cutoff of 450 eV)^75^ were used to describe the valence electrons: 11 electrons of Cu (3d10 4s^1^), 10 electrons of Ni (3d^8^4s^2^). The relaxation of the atomic positions in the supercell occurred until the forces were smaller than 0.01 eV Å^−1^ (EDIFFG = −0.01). Clusters are positioned in a 20 × 20 × 20 Å simulation box with periodic boundary conditions, and a vacuum layer of more than 8 Å was included to eliminate interactions between nanoparticles and their periodic images. To aid convergence in metallic systems, Methfessel–Paxton smearing was applied with a sigma value of 0.01 eV.^76^

Although the 55-atom icosahedral motif is well established in the literature and is frequently adopted as a reference structure for monometallic clusters such as Cu_55_, the use of the ABCluster global optimization framework in this work was not intended to rediscover a known geometry. Rather, it was employed to rigorously confirm the true global minimum structures of both Cu_55_ and Cu_13_@Ni_42_ within a unified and self-consistent computational framework. The energetic ordering of cluster isomers is known to be sensitive to the choice of exchange–correlation functional, spin treatment, relaxation criteria, and numerical parameters, and therefore reliance on literature-optimized structures may introduce unintended methodological bias. This consideration is particularly critical for the bimetallic Cu_13_@Ni_42_ cluster, where multiple competing chemical orderings, segregation patterns, and low-lying isomers exist on a highly rugged potential energy surface. The ABCluster-DFT approach allows for an unbiased exploration of this landscape, verifies the thermodynamic stability of the core–shell configuration relative to alternative arrangements, and ensures that the catalytic trends discussed in this study originate from genuine structure–property relationships rather than assumed geometries. Consequently, global optimization provides a robust structural foundation for meaningful comparison of adsorption energetics, reaction thermochemistry, and electronic properties across the two catalyst systems.

### 2.3. Global structure optimization and convergence tests

The global minimum structures of the Cu_55_ and Cu_43_Ni_12_ clusters were identified using the artificial bee colony (ABC) algorithm as implemented in the ABCluster program. In the global optimization, the population size was set to 500 trial structures, the maximum number of generations to 1000, and the scout limit to 5. These parameters implicitly determine the numbers of employed and onlooker bees and the iteration limits, which are managed internally by the ABCluster algorithm and are not explicitly user-defined. With this setup, more than 50 000 candidate structures were sampled in total, ensuring exhaustive exploration of the potential energy surface and reliable identification of the global minimum configurations.

To ensure the numerical reliability of the DFT calculations, convergence tests were performed with respect to plane-wave cutoff energy and simulation cell size. A cutoff energy of 450 eV was found to converge total energies within 1 meV per atom. The simulation cell was increased up to 20 × 20 × 20 Å, beyond which total-energy variations were less than 0.5 meV per atom, confirming negligible interactions between periodic images. Although *Γ*-point sampling is sufficient for these zero-dimensional cluster systems due to the large vacuum region, additional *k*-point sampling was also tested and produced no significant changes in total energies or atomic forces. These settings were therefore adopted for all production calculations.

### 2.4. Energetics analysis

#### 2.4.1. Binding energy

The binding energy per atom (*E*_b_), which is related to the stability of nanoclusters, (*E*_b_) can be calculated as follows:^77^1
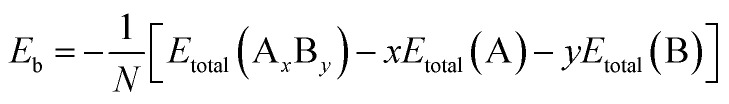
where *x* and *y* are the numbers of A and B atoms; *E*_total_ (A) and *E*_total_ (B) are the electronic energies of single atoms, and *N* is the total number of atoms (*N* = *x* + *y*).

The total energy of a nanoalloy is denoted by *E*_total(A_*x*_B_*y*_)_, *E*_total_ (A_*x*+*y*_) and *E*_total_ (B_*x*+*y*_) are the energies of the pure nanoparticles with the same size (*x* + *y*) as A_*x*_B_*y*_, and *x* and *y* are the number of atoms of metal A and B, respectively.

#### 2.4.2. Adsorption energy

For modeling and studying possible reaction pathways, the adsorption energy is calculated by the formula:

2 *E*_ads_ = *E*_(system)_ − [*E*_Cluster_ + *E*_Adsorbate_]

where *E*_(system)_, is the total energy of the cluster with the adsorbed species, *E*_Cluster_ is the total energy of the cluster, and *E*_Adsorbate_ is the total energy of the isolated adsorbed species. More negative values indicate stronger adsorption. Meaning a negative *E*_ads_ value indicates an exothermic and energetically favorable adsorption process.

## 3. Results and discussion

**Fig. 2:**
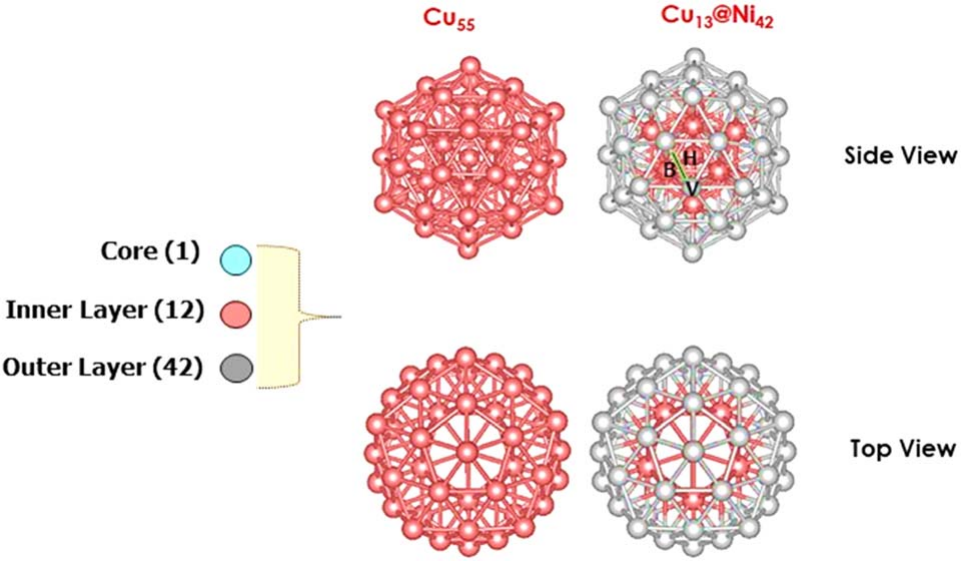
Optimized geometries of Cu_55_, and core–shell Cu_13_@Ni_42_. And the possible adsorption sites: H: hollow, B: bridge, V:atop.

The conversion of CO_2_ to formic acid through hydrogenation involves a sequence of three fundamental steps: (1) the reactant is first adsorbed onto the surface of the electrocatalyst, (2) electron/proton transfer to the reactant, and (3) release of the product from the electrocatalyst surface.^78^ To initiate our theoretical investigation, we conducted comprehensive structural optimizations of two distinct nanoalloy models: a monometallic copper (Cu_55_) and a bimetallic core–shell configuration consisting of a copper core encapsulated by a nickel shell (Cu_13_@Ni_42_). The obtained optimized geometries are presented in Fig. 2 and serve as the foundational models for subsequent analysis of their catalytic behavior.

### 3.1. Catalytic performance of nanoalloys

#### 3.1.1. Monometallic Cu_55_

**Fig. 3:**
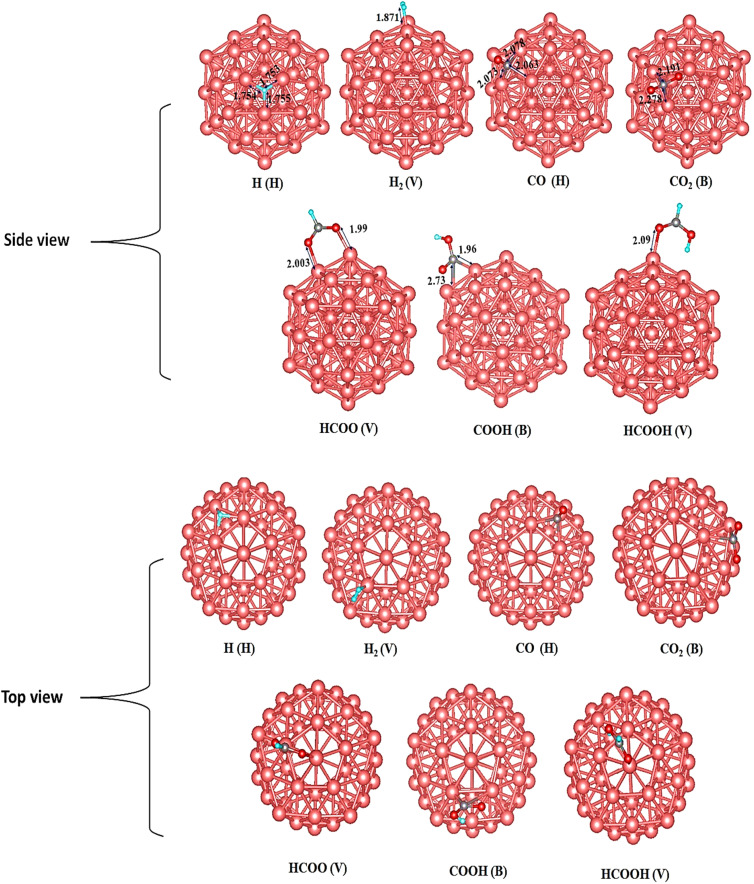
Most stable adsorption configurations on Cu_55_ of intermediates (distances in Å). As indicated by orange, gray, green, and red balls, Cu, C, H, and O atoms, respectively. V, H, and B represents the top, hollow, and bridge sites.

The adsorption energies and configurations obtained from these investigations are summarized in Table 1 and visually depicted in Fig. 3.

**Table 1:** Adsorption energies (eV) of intermediates on Cu_55_ cluster, V, H, and B represents the top, hollow, and bridge sites

Species	Site	Bond parameters (Å)	E_ads_ (eV)
H	H	*d*_M–H_ = 1.755, 1.754, 1.753	−2.677
H_2_	V	*d*_M–H_ = 1.871	−4.660
CO	H	*d*_M–C_ = 2.073, 2.063, 2.078	−1.094
CO_2_	B	*d*_M–C_ = 2.278, 2.191	−0.102
HCOO	V–V	*d*_M–O_ = 1.99, 2.003	−3.297
COOH	B	*d*_M–C_ = 1.96, 2.73	−1.952
HCOOH	V	*d*_M–O_ = 2.09	−0.705

To identify the thermodynamically favored adsorption configurations, three adsorption modes were systematically investigated for each adsorbate. Density Functional Theory (DFT) calculations reveal that the hydrogen atom preferentially adsorbs at the hollow site, where it coordinates with three Cu atoms. The calculated Cu–H bond lengths are 1.755, 1.754, and 1.753 Å, with a corresponding adsorption energy of −2.677 eV, indicating a stable interaction. For molecular hydrogen (H_2_), the most favorable adsorption site is the top site, characterized by a Cu–H distance of 1.871 Å and a notably high adsorption energy of −4.660 eV, suggesting strong chemisorption. The CO intermediate exhibits the strongest binding at the hollow site, with the carbon atom coordinated to three Cu atoms. The Cu–C bond lengths are 2.073, 2.063, and 2.078 Å, yielding an adsorption energy of −1.094 eV. In contrast, CO_2_ preferentially adsorbs at the bridge site, where the carbon atom interacts with two Cu atoms. The corresponding Cu–C bond lengths are 2.278 and 2.191 Å. The adsorption energy is calculated to be −0.102 eV, and the adsorbed CO_2_ molecule undergoes a significant deviation from linearity, indicative of activation upon adsorption. The HCOO intermediate exhibits the strongest adsorption at the top site, where it binds through both oxygen atoms to two Cu atoms. The Cu–O bond lengths are 1.990 Å and 2.003 Å, and the adsorption energy is −3.297 eV. The COOH intermediate favors adsorption at the bridge site, with the carbon atom coordinated to two Cu atoms at distances of 1.960 Å and 2.730 Å, resulting in an adsorption energy of −1.952 eV. For formic acid (HCOOH), the top site is the most energetically preferred, where adsorption occurs *via* the oxygen atom. The Cu–O bond length is 2.090 Å, and the corresponding adsorption energy is −0.705 eV.

**Fig. 4:**
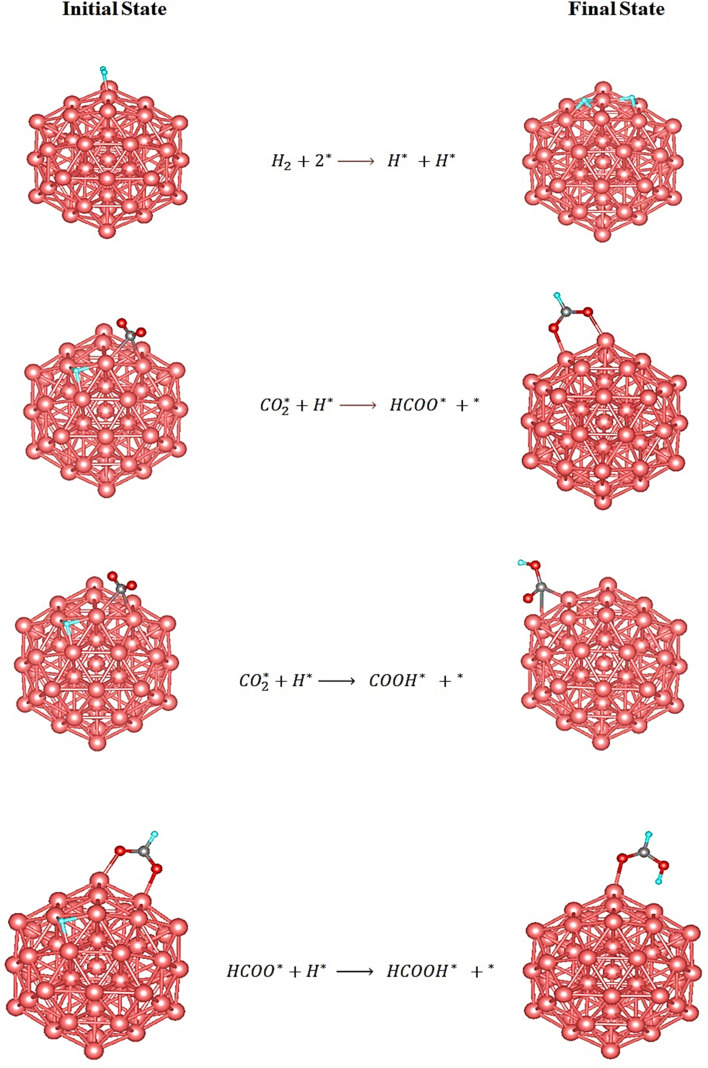
The initial and the final state of elementary steps of HCOOH production over Cu_55_ cluster.

The adsorption geometries of the reaction intermediates modeled in this study are in excellent agreement with those reported in previous DFT study,^79^ supporting the reliability of our computational methodology and suggesting that the observed binding preferences are intrinsic to the Cu surface–intermediate interactions. Upon identifying the most thermodynamically stable adsorption geometries for each intermediate, the subsequent step involves exploring the possible hydrogenation pathways on the Cu_55_ cluster. The hydrogenation of CO_2_ necessitates the initial dissociation of molecular hydrogen (H_2_) into atomic hydrogen (H*). As illustrated in Fig. 4, the first elementary step involves H_2_ adsorption at the top site of a surface Cu atom, followed by heterolytic cleavage to yield two surface-bound H atoms. Following this, coadsorption of 
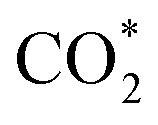
 and H* occurs on the Cu_55_ surface, enabling two competing hydrogenation pathways. In the first reaction pathway, an adsorbed hydrogen atom (H*) attacks the carbon atom of adsorbed 
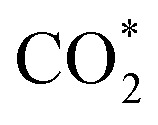
 to form the formate intermediate (HCOO*). Alternatively, in the second pathway, H* binds to an oxygen atom of 
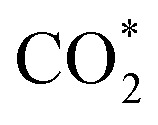
, leading to the formation of the COOH* intermediate. However, this route is thermodynamically less favorable, as the adsorption energy of HCOO* is −3.297 (1.345 eV lower than that of COOH*), indicating greater stability. Further hydrogenation of the HCOO* species yields formic acid (HCOOH*). Significantly, the weak adsorption energy of −0.705 eV on the Cu_55_ cluster implies that formic acid can readily desorb from the surface.

#### 3.1.2. Bimetallic Cu_13_@Ni_42_

**Fig. 5:**
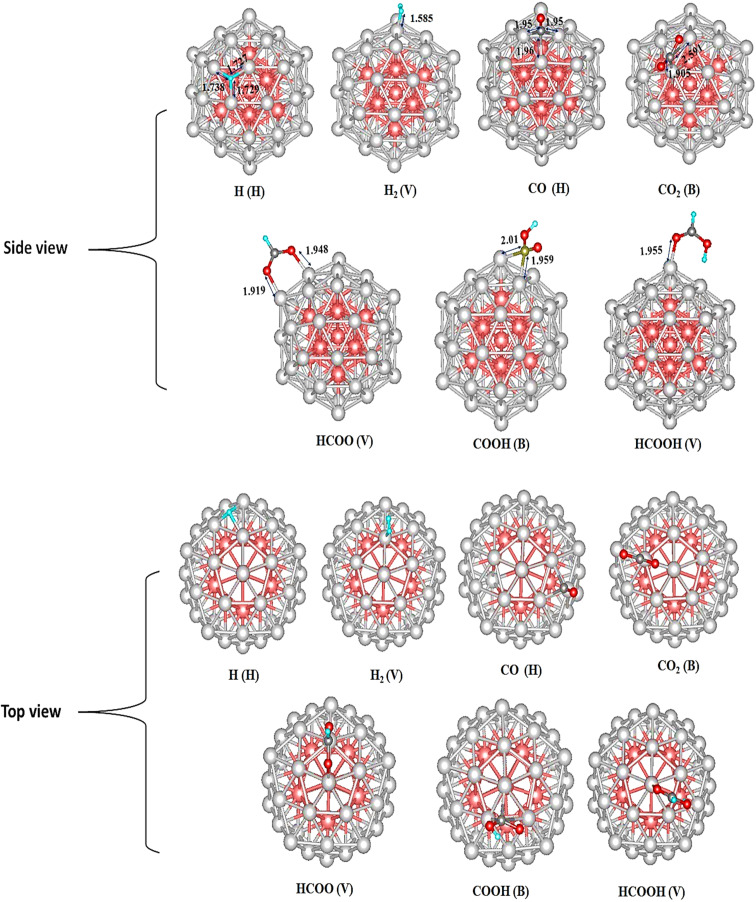
Most stable adsorption configurations on bimetallic core–shell Cu_13_@Ni_42_ of intermediates (distances in Å). As indicated by gray, green, and red balls, C, H, and O atoms, respectively. V, H, and B represent the top, hollow, and bridge sites.

Details on the bond parameters and adsorption energies of Cu_13_@Ni_42_ are presented in Table 2, with Fig. 5 illustrating the most stable adsorption configurations of the intermediates.

**Table 2:** Adsorption energies (eV) of intermediates on Cu_13_@Ni_42_ cluster, V, H, and B represents the top, hollow, and bridge sites

Species	Site	Bond parameters (Å)	E_ads_ (eV)
H	H	*d*_M–H_ = 1.727, 1.738, 1.729	−2.862
H_2_	V	*d*_M–H_ = 1.585	−5.002
CO	H	*d*_M–C_ = 1.95, 1.95, 1.96	−3.745
CO_2_	B	*d*_M–C_ = 2.591, 1.905	−0.924
HCOO	V	*d*_M–O_ = 1.919, 1.948	−3.641
COOH	B	*d*_M–C_ = 2.01, 1.959	−2.561
HCOOH	V	*d*_M–O_ = 1.955	−0.927

To investigate the potential hydrogenation pathways on the Cu_13_@Ni_42_ core–shell cluster, it is first necessary to determine the most stable adsorption configurations of the reaction intermediates. Based on our DFT calculations, the hydrogen atom exhibits a clear preference for the hollow site, where it is coordinated to three Ni atoms. The calculated Ni–H bond distances are 1.727, 1.738, and 1.729 Å, and the corresponding adsorption energy is −2.862 eV. As for H_2_ molecule, the best site was found to be the top site, with a Cu–H distance of 1.585 Å. It is worth mentioning that compared to Cu_55_, H_2_ adsorption energy on the bimetallic Cu_13_@Ni_42_ cluster is −5.002 eV, more than that of Cu_55_ by 1.075 eV. This is indicative of a stronger interaction between Ni and H_2_. Among all investigated configurations for the CO intermediate, the hollow site yields the most stable adsorption, with the carbon atom coordinated to three Ni atoms. The Ni–C bond lengths are 1.950, 1.950, and 1.960 Å, and the corresponding adsorption energy is −3.745 eV. This is 2.651 eV more exothermic than on Cu_55_, signifying substantially enhanced binding of CO on the bimetallic cluster. On the other hand, CO_2_ preferentially adsorbs at the bridge site, where its carbon atom forms bonds with two Ni atoms, exhibiting Ni–C distances of 2.591 and 1.905 Å. The adsorption energy is −0.924 eV, which is 0.821 eV more exothermic than on the Cu_55_ surface. Upon adsorption, the CO_2_ molecule undergoes a structural distortion from its linear configuration to a bent geometry. The HCOO* intermediate adsorbs most favorably at the top site, binding *via* both oxygen atoms to two Ni atoms. The Ni–O bond lengths are 1.919 and 1.948 Å, and the adsorption energy is −3.641 eV, indicating strong interaction with the Ni surface. Similarly, the COOH* intermediate prefers the bridge site, where the carbon atom is bonded to two Ni atoms. The corresponding Ni–C bond distances are 2.010 and 1.959 Å, and the adsorption energy is −2.561 eV. For formic acid (HCOOH*), the top site is again the most favorable, with one oxygen atom coordinated to a Ni atom at a bond length of 1.955 Å. The associated adsorption energy is −0.927 eV, indicating relatively weak binding, which could facilitate product desorption.

In nanoclusters, catalytic activity is inherently site dependent due to the presence of atoms with different coordination environments. In the Cu_55_ and Cu_13_@Ni_42_ clusters considered here, vertex and edge atoms are markedly undercoordinated compared with facet-like atoms, leading to localized electronic states closer to the Fermi level and enhanced reactivity. Our adsorption calculations indicate that CO_2_ and key hydrogenated intermediates preferentially bind to these low-coordination sites, particularly at vertex and edge positions, rather than on more saturated facet sites. This behavior reflects the higher availability of unsaturated metal orbitals and increased local density of states associated with undercoordinated atoms. In the Cu_13_@Ni_42_ core–shell cluster, Ni incorporation further modifies the reactivity of surface Cu sites through electronic ligand effects and lattice strain, shifting the Cu d states toward the Fermi level and strengthening interactions at reactive edge and vertex sites. As a result, these sites play a dominant role in stabilizing formate-related intermediates and driving the reaction pathway toward formic acid, while more highly coordinated facet sites contribute less to catalytic turnover. This site-specific perspective clarifies how local geometry and electronic structure jointly determine activity and selectivity on Cu–Ni nanoclusters.

**Fig. 6:**
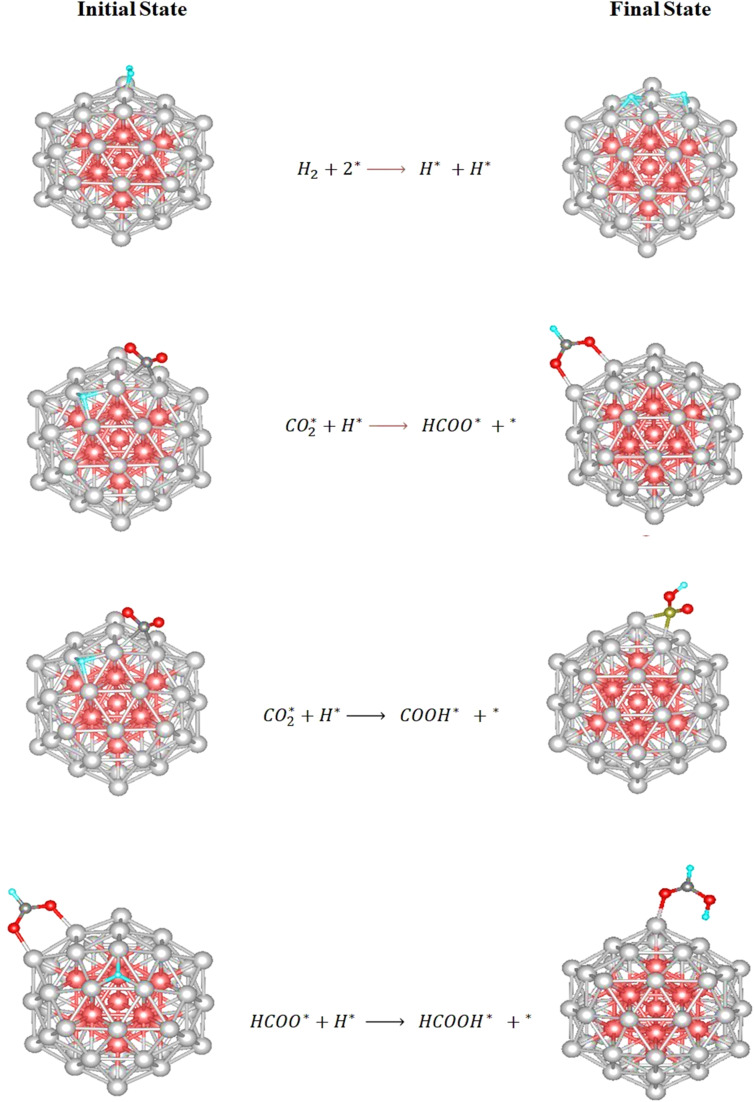
The initial and the final state of elementary steps of HCOOH production over bimetallic core–shell Cu_13_@Ni_42_.

The DFT-calculated adsorption configurations and energetics on the Cu_13_@Ni_42_ cluster are consistent with a previously published theoretical study.^79^ With the stable adsorption structures identified, we proceeded to explore the possible reaction pathways for CO_2_ hydrogenation on this surface. The initial step involves H_2_ dissociation into atomic hydrogen. As shown in Fig. 6, the H_2_ molecule adsorbs at the top site of a Ni atom and subsequently cleaves into two surface-bound H atoms. Following this, coadsorption of 
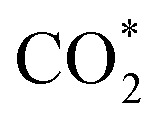
 and H* on the Cu_13_@Ni_42_ surface leads to two competing hydrogenation pathways. In the first pathway, the H* atom attacks the carbon atom of the adsorbed 
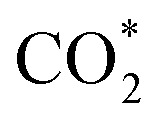
, leading to the formation of the formate intermediate (HCOO*). In the alternative pathway, H* binds to one of the oxygen atoms of 
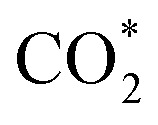
, forming the carboxyl intermediate (COOH*). However, this pathway is thermodynamically less favorable, as the adsorption energy of HCOO* is 0.886 eV more exothermic than that of COOH*. The final step involves hydrogenation of HCOO* by an additional H* atom to form formic acid (HCOOH*). Given its relatively low adsorption energy of −0.927 eV, HCOOH is expected to desorb readily from the catalyst surface, completing the CO_2_ hydrogenation sequence.

### 3.2. Thermochemistry

**Fig. 7:**
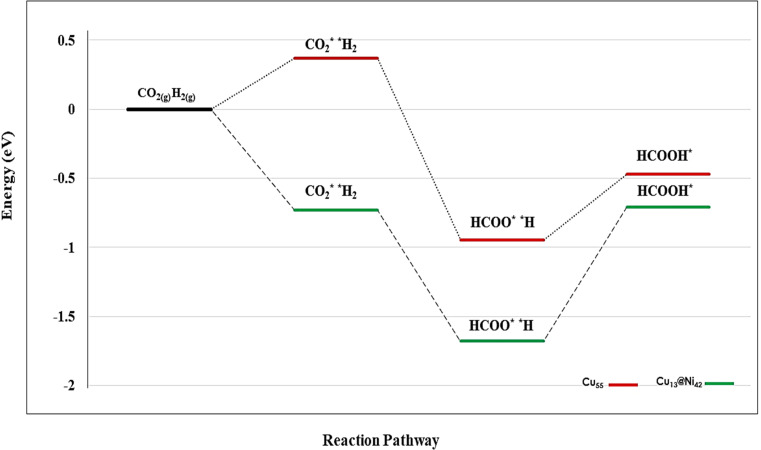
Thermochemistry of the CO_2_ conversion to formic acid over Cu_55_ (red) and bimetallic Cu_13_@Ni_42_ (green).

From a thermodynamic perspective (as shown in Fig. 7), the proposed reaction pathway proceeds as follows: the CO_2_ electroreduction (CO_2_ER) begins with the co-adsorption of CO_2_ and H_2_ molecules on the Cu_55_ cluster. The H_2_ molecule dissociates into two hydrogen atoms, one of which approaches the CO_2_ molecule, leading to the formation of the HCOO* intermediate (Δ*E* = −0.984 eV). Subsequently, the second hydrogen atom binds to the oxygen atom of HCOO, resulting in the formation of formic acid on the surface (Δ*E* = −0.470 eV). The catalytic activity of the Cu_13_@Ni_42_ cluster can be assessed by examining the relative energies of the key steps involved in the CO_2_ reduction reaction (CO_2_RR) toward formic acid, as illustrated in Fig. 7. As described earlier, the reaction initiates with the co-adsorption of CO_2_ and H_2_ molecules on the Cu_13_@Ni_42_ surface. Upon dissociation of H_2_, one hydrogen atom migrates toward the CO_2_ molecule, resulting in the formation of the HCOO intermediate (Δ*E* = −1.679 eV). The final step involves the addition of a second hydrogen atom to the oxygen atom of HCOO, leading to the formation of formic acid (HCOOH) on the surface (Δ*E* = −0.709 eV). According to previous DFT studies,^80^ the hydrogenation of the formate intermediate to form formic acid (HCOO* → HCOOH*) is identified as the rate-determining step. Based on these findings, the bimetallic core–shell Cu_13_@Ni_42_ cluster is expected to exhibit superior catalytic performance compared to its monometallic Cu_55_ counterpart.

All DFT calculations in this work were performed at 0 K, which is a common approximation for investigating adsorption energetics and reaction trends on catalytic surfaces and clusters. While finite-temperature effects, including vibrational, rotational, and translational entropy contributions, can influence absolute free energies and reaction rates, such effects often partially cancel when comparing similar intermediates and competing pathways on the same catalyst. Consequently, 0 K reaction energetics remain reliable for establishing relative stability trends, identifying preferred intermediates, and rationalizing selectivity. In particular, the key conclusions drawn here-namely the enhanced stabilization of formate-related intermediates and the suppression of competing pathways on the Cu_13_@Ni_42_ cluster-are primarily governed by electronic-structure effects that are expected to persist under practical reaction conditions. Nevertheless, incorporating full free-energy corrections and explicit kinetic barriers at finite temperatures and pressures would provide a more quantitative assessment of reaction feasibility and is a valuable direction for future work.

### 3.3. Selectivity toward formic acid and competing reaction pathways

In addition to the formic-acid formation pathway examined here, CO_2_ hydrogenation can in principle proceed *via* competing routes leading to CO (reverse water–gas shift) or more deeply hydrogenated products such as methanol. These alternative pathways require stabilization of intermediates such as *CO, *CHO, or *CH_3_O, which are known to bind strongly on Ni-rich surfaces. However, our electronic-structure and adsorption analyses indicate that the Cu_13_@Ni_42_ core–shell cluster exhibits a balanced interaction strength with reaction intermediates: Cu–Ni d–d hybridization shifts the effective d-band center toward the Fermi level, enhancing stabilization of formate-like species (*HCOO/*COOH), while avoiding excessively strong binding of CO-like intermediates. As a result, pathways involving CO accumulation or deep hydrogenation are thermodynamically disfavored relative to the formic-acid route. This selective stabilization explains why the Cu_13_@Ni_42_ cluster preferentially promotes formic acid formation while suppressing undesired byproducts, highlighting the role of controlled electronic-structure modulation in determining product selectivity.

### 3.4. Electronic structure and density of states

**Fig. 8:**
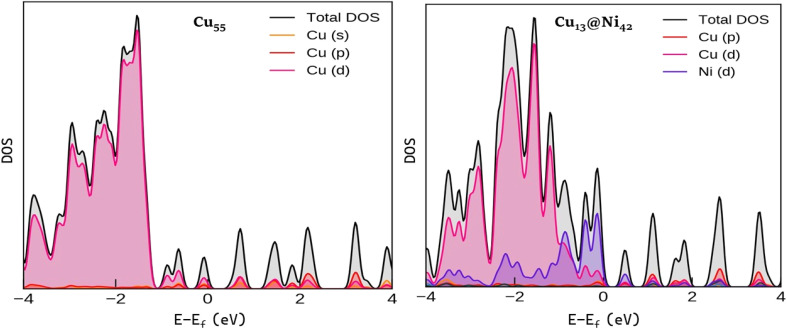
Density of states (eV) of Cu_55_ and Cu_13@_Ni_42_. The Fermi energy level is set to 0 eV. The DOS features near the Fermi level correlate directly with the stabilization of reaction intermediates discussed in Section (3.1 Catalytic performance of nanoalloys).

To gain a more comprehensive understanding of the electronic structure of these nanoparticles, density of states (DOS) calculations were performed, as shown in Fig. 8. The d-band center in Cu, which has a direct relationship to catalysis, is completely filled and is lower than the Fermi level.^81,82^ Additionally, as demonstrated in our previous work^83^ the nearly identical spin-up and spin-down electronic states in the Cu_55_ cluster confirm its nonmagnetic nature, as reflected by its net magnetic moment of 0 µβ. In other words, substituting Cu atoms with Ni significantly alters the electronic structure, as reflected by the magnetic moment of Ni_55_, which is 41.05 µβ. Notably, the d-orbitals play a crucial role in the bonding interactions and can enhance the structural stability of bimetallic nanoparticles. There is also observable overlap between the 3d orbitals of Cu and Ni, suggesting that both composition and geometry influence the position of the d-band center.^84^ This highlights the intrinsic difference in the electronic configurations of Cu and Ni, where Ni contributes to localized magnetic moments and partially filled d-states that can influence reactivity. In the case of the Cu_43_@Ni_12_ cluster, the density of states (DOS) plot clearly reveals the overlap between the 3d orbitals of Cu and Ni atoms within the bonding region. This orbital hybridization facilitates stronger metal–metal interactions and can modify both the electronic and catalytic properties of the nanoalloy. Overall, these observations suggest that the electronic structure, magnetic behavior, and stability of Cu–Ni nanoparticles are highly sensitive to both composition and atomic arrangement. The position and character of the d-band serves as a key descriptor for predicting and tuning their catalytic performance.

The DOS for the Cu_55_ cluster and the core–shell bimetallic Cu_13_@Ni_42_ cluster are shown in Fig. 8. Both systems exhibit finite DOS at the Fermi level, confirming their metallic character and the potential for electron exchange with adsorbates; however, pronounced differences in spectral shape, bandwidth, and orbital contributions reveal that Cu–Ni alloying substantially alters the electronic structure. For monometallic Cu_55_, the electronic states between approximately −4 and 0 eV are dominated by Cu 3d orbitals, with only minor Cu s and p contributions. The Cu d band is largely filled and characterized by several sharp peaks mainly between −3 and −1 eV, reflecting the discrete energy levels of a finite cluster and indicating that the d-band center lies well below the Fermi level, which is generally associated with relatively weaker adsorbate interactions. In contrast, the Cu_13_@Ni_42_ core–shell cluster displays pronounced band broadening, a redistribution of Cu d states toward the Fermi level, and the emergence of Ni d states that strongly overlap with Cu d states in the −3 to 0 eV region, resulting in a higher DOS intensity near the Fermi level. These features are clear signatures of strong Cu–Ni d–d hybridization at the core–shell interface, accompanied by partial charge redistribution driven by differences in electronegativity and d-band filling, as well as additional strain effects induced by lattice mismatch. Importantly, these electronic modifications have direct catalytic implications: the increased DOS near the Fermi level facilitates charge transfer, the upward shift of the effective d-band center enhances stabilization of key hydrogenated intermediates, and Cu–Ni hybridization allows adsorption strengths to be tuned without excessively strong binding of CO-like species. As a result, the Cu_13_@Ni_42_ cluster exhibits more favorable adsorption energetics and reaction thermochemistry along the CO_2_ hydrogenation pathway to formic acid compared with Cu_55_, demonstrating that the improved catalytic performance originates from controlled electronic-structure modulation rather than from CO_2_ activation alone.

The electronic origin of the enhanced CO_2_ activation on bimetallic clusters can be further clarified through Bader charge analysis. In the monometallic Cu_55_ cluster, electron density is redistributed from the core toward the surface, with the central atom, the 12 inner-shell atoms, and the 42 outer-shell atoms carrying net charges of −0.07, +0.95, and −0.88 e, respectively, indicating intrinsic charge polarization even in the absence of alloying. Upon Ni incorporation, the Cu_13_Ni_42_ cluster exhibits enhanced charge transfer from the core atom to the middle-layer atoms, reflecting stronger electronic redistribution driven by Cu–Ni interactions. This increased charge separation modifies the local electronic environment of surface atoms, improving their ability to donate and accept electron density during CO_2_ adsorption and activation. Combined with the pronounced Cu–Ni d–d hybridization observed in the density of states, this quantitative charge redistribution provides direct evidence that ligand effects induced by Ni play a key role in tuning the electronic structure and reactivity of the cluster. As a result, the bimetallic Cu_13_Ni_42_ system exhibits more favorable electronic properties than monometallic Cu_55_, consistent with its enhanced stabilization of hydrogenated intermediates and improved catalytic performance.^83^

### 3.5. Practical relevance

Although the present study is theoretical in nature, its predictions are consistent with experimentally observed trends reported for Cu–Ni bimetallic catalysts in CO_2_ hydrogenation and related reactions. Experimental studies have shown that incorporation of Cu into Ni-based catalysts significantly alters the surface electronic and geometric structure, leading to changes in CO_2_ activation and product selectivity compared with monometallic systems. For example, well-dispersed Cu–Ni bimetallic nanoparticles supported on γ-Al_2_O_3_ exhibit modified CO_2_ hydrogenation activity and enhanced CO selectivity relative to monometallic counterparts, which has been correlated to restructuring of the core–shell architecture under reaction conditions and altered surface electronic states observed by ambient pressure X-ray photoelectron spectroscopy (AP-XPS) and *in situ* spectroscopy.^85^ Near-ambient pressure X-ray photoelectron spectroscopy studies of model Ni/Cu surfaces further demonstrate the formation of formate intermediates (*HCOO) during CO_2_ hydrogenation on Ni–Cu surfaces, highlighting how electronic interactions in the alloy influence intermediate binding and reaction pathways.^86^ These observations qualitatively support our finding that Cu–Ni d–d hybridization and core–shell architectures stabilize key hydrogenated intermediates while avoiding excessively strong binding of CO-like species. The present results further provide clear guidance for experimental validation: size-controlled Cu–Ni core–shell nanoclusters or nanoparticles could be synthesized using colloidal or vapor-phase methods, followed by CO_2_ hydrogenation measurements under mild conditions. *In situ* or *operando* spectroscopic techniques, such as infrared or Raman spectroscopy, could be employed to probe the stabilization of reaction intermediates predicted here. Systematic variation of Cu/Ni composition and particle size would allow direct testing of the proposed structure–property–reactivity relationships, thereby bridging theoretical predictions and experimental catalyst design.

### 3.6. Stability considerations and implications for catalytic turnover

Beyond intrinsic activity and selectivity, catalyst stability and turnover frequency are essential metrics for practical applications. Supported bimetallic catalysts have been shown to enhance catalyst stability relative to monometallic systems, with alloy formation often mitigating deactivation pathways such as sintering and carbon deposition.^87^ Although explicit simulations of long-term stability phenomena such as sintering, oxidation, or structural reconstruction under reaction conditions were not performed in this study, several features of the bimetallic Cu_13_@Ni_42_ cluster suggest improved robustness relative to monometallic Cu_55_. For example, experimental work on Cu–Ni systems demonstrates that Ni incorporation into Cu catalysts can improve stability and regenerability in hydrodeoxygenation reactions.^88^ The strong Cu–Ni interactions, pronounced charge redistribution, and stabilization of the core–shell architecture indicate enhanced resistance to structural degradation, which is consistent with experimental observations that alloying Cu with Ni can improve thermal and chemical stability by suppressing deactivation mechanisms.^89^

With respect to catalytic rates, quantitative turnover frequencies require detailed kinetic modeling that includes activation barriers, surface coverages, and finite-temperature effects, which are necessary to connect thermodynamic trends to measurable reaction rates. Current experimental and theoretical literature highlights that while bimetallic catalysts often display improved intrinsic activity and selectivity trends, accurate TOFs depend on both detailed kinetic data and reaction mechanism analysis.^88^ While such calculations are beyond the present scope, the thermodynamic favorability of key reaction steps and the selective stabilization of formate-related intermediates on Cu_13_@Ni_42_ strongly suggest enhanced intrinsic activity compared with Cu_55_. These results provide a solid foundation for future kinetic modeling and experimental validation aimed at quantifying TOFs and long-term catalyst durability.

## 4. Conclusions

In this work, a comprehensive DFT investigation was carried out to examine the structural, electronic, and catalytic properties of monometallic Cu_55_ and bimetallic core–shell Cu_13_@Ni_42_ nanoclusters for the hydrogenation of CO_2_ to formic acid. Global optimization using the artificial bee colony (ABC) algorithm enabled the identification of the most stable geometries, which served as the foundation for evaluating adsorption behavior and reaction pathways. The optimized structures revealed the inherent stability of the 55-atom icosahedral framework and highlighted the significant structural changes arising from Ni incorporation in the core–shell configuration. The selective stabilization of formate intermediates over CO-like species suggests that Cu–Ni core–shell clusters may inherently suppress competing CO and methanol pathways.

The adsorption studies demonstrated that both clusters can activate and bind CO_2_ through bending of the molecular geometry, a key requirement for initiating hydrogenation. However, the Cu_13_@Ni_42_ cluster consistently exhibited more exothermic adsorption energies for all intermediates, including CO_2_*, H*, CO*, HCOO*, and COOH*. This enhanced binding strength points to a stronger interaction between the Ni-rich surface and the adsorbates. Importantly, for both catalysts, the hydrogenation reaction was found to proceed preferentially through the formate (HCOO*) pathway rather than the carboxyl (COOH*) route, consistent with the relative stability of the intermediates.

Thermochemical analysis revealed substantial differences in catalytic performance between the two clusters. The formation of the HCOO* intermediate is significantly more exothermic on Cu_13_@Ni_42_ (−1.679 eV) than on Cu_55_ (−0.984 eV), indicating a more favorable first hydrogenation step. Similarly, the subsequent hydrogenation to HCOOH is also more energetically favorable on Cu_13_@Ni_42_, confirming that the bimetallic system offers a more efficient reaction pathway. In both cases, the relatively weak adsorption of the final product, formic acid, suggests that it can desorb readily from the catalyst surface, an essential feature for sustained catalytic turnover.

Electronic structure analysis provided additional insight into the superior activity of the bimetallic catalyst. Density of states calculations revealed substantial modification of electronic properties upon Ni incorporation, including changes in the d-band characteristics that influence both adsorption and reactivity. The hybridization between Cu and Ni d-states enhances the interaction with adsorbates and stabilizes key intermediates. Collectively, these findings establish that the Cu_13_@Ni_42_ core–shell nanocluster outperforms its monometallic Cu_55_ counterpart in CO_2_ activation, intermediate stabilization, and overall thermodynamic favorability. This study highlights the potential of Cu–Ni nanoalloys as promising candidates for selective CO_2_-to-formic acid conversion and provides valuable insights for the future design of efficient bimetallic electrocatalysts. Future work combining experimental measurements and advanced kinetic modeling will be necessary to evaluate long-term stability and turnover frequencies under realistic reaction conditions.

## Conflicts of interest

There are no conflicts to declare.

## Data Availability

The data supporting this article have been included as part of the main manuscript.
